# Congenital renal arteriovenous malformation presenting with gross hematuria after a routine jog: a case report

**DOI:** 10.1186/1752-1947-8-65

**Published:** 2014-02-20

**Authors:** Erin L Dames, Lay Guat Ng, Kiang Hiong Tay

**Affiliations:** 1 Department of Diagnostic Radiology, Singapore General Hospital, Outram Road, Singapore 169608, Singapore; 2 Department of Urology, Singapore General Hospital, Outram Road, Singapore 169608, Singapore

**Keywords:** Arteriovenous malformation, Hematuria, Computed X-ray tomography, Angiography, Therapeutic embolization

## Abstract

**Introduction:**

Congenital renal arteriovenous malformations are abnormal communications between the intrarenal venous and arterial systems. An unusual cause of massive gross hematuria and an even rarer cause of hemodynamically significant anemia, its presentation remains variable from incidental imaging findings to severe hypertension and congestive heart failure.

**Case presentation:**

We present a case of a 44-year-old Chinese man with no personal or familial history of bleeding diasthesis that presented with gross hematuria leading to clot retention after routine physical activity.

**Conclusions:**

We have presented this case in an effort to highlight the possibility of this entity as a cause of acute upper urinary tract hemorrhage and the need for a computed tomography angiogram to clinch the diagnosis.

## Introduction

Congenital renal arteriovenous malformations (AVMs) are abnormal communications between the intrarenal venous and arterial system. Such anomalies in the renal vasculature are rare causes of gross hematuria. A significant number of patients with renal AVMs are hypertensive [1], and its clinical presentation is variable, ranging from incidental finding in an asymptomatic patient to congestive cardiac failure due to high output from a giant AVM. Its classical presentation is that of gross hematuria, occasionally associated with the passage of blood clots and flank pain. We present a case of a renal AVM presenting as gross hematuria after routine physical activity with multiple episodes of clot retention.

## Case presentation

A 44-year-old Chinese man presented to the emergency department with the chief complaint of gross hematuria with blood clots, which he had noticed after a routine morning run. He had a known medical background of hypertension. He was a nonsmoker with a moderately active lifestyle, jogging regularly. He had no personal or familial history of bleeding disorders.

The initial physical examination was unremarkable, and the bedside ultrasound examination showed a large amount of clots in the urinary bladder, without any evidence of hydronephrosis. Initial investigations showed no evidence of anemia, infection, coagulopathy or renal impairment. Initial hematologic and biochemical investigations showed a hemoglobin of 16.1g/dL, hematocrit of 47.3%, creatinine level of 83umol/L, urea of 4.2mmol/L, activated partial thromboplastin time of 31.4 seconds and prothrombin time of 10 seconds.

He was catheterized, draining gross hematuria with a substantial amount of clots. A manual bladder clot evacuation was performed, after which he was started on continuous bladder irrigation, intravenous antibiotic coverage and subsequently admitted to the general inpatient ward.

**Figure 1:**
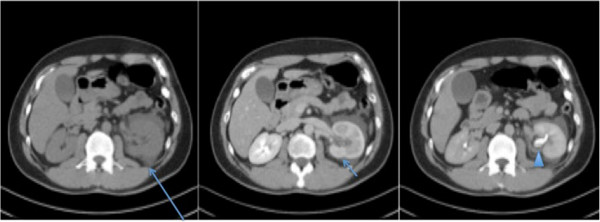
**Axial computed tomography image in the plain, nephrographic and pyelographic phases.** A large amount of perinephric fluid is seen on the left side (long arrow in first panel). Delayed enhancement of the left kidney with delayed excretion of contrast is apparent (short arrow in second panel), but no solid mass or urinary calculus is seen. Dependent material is seen in the nondilated collecting system of the left kidney consistent with blood (arrowhead in third panel).

**Figure 2:**
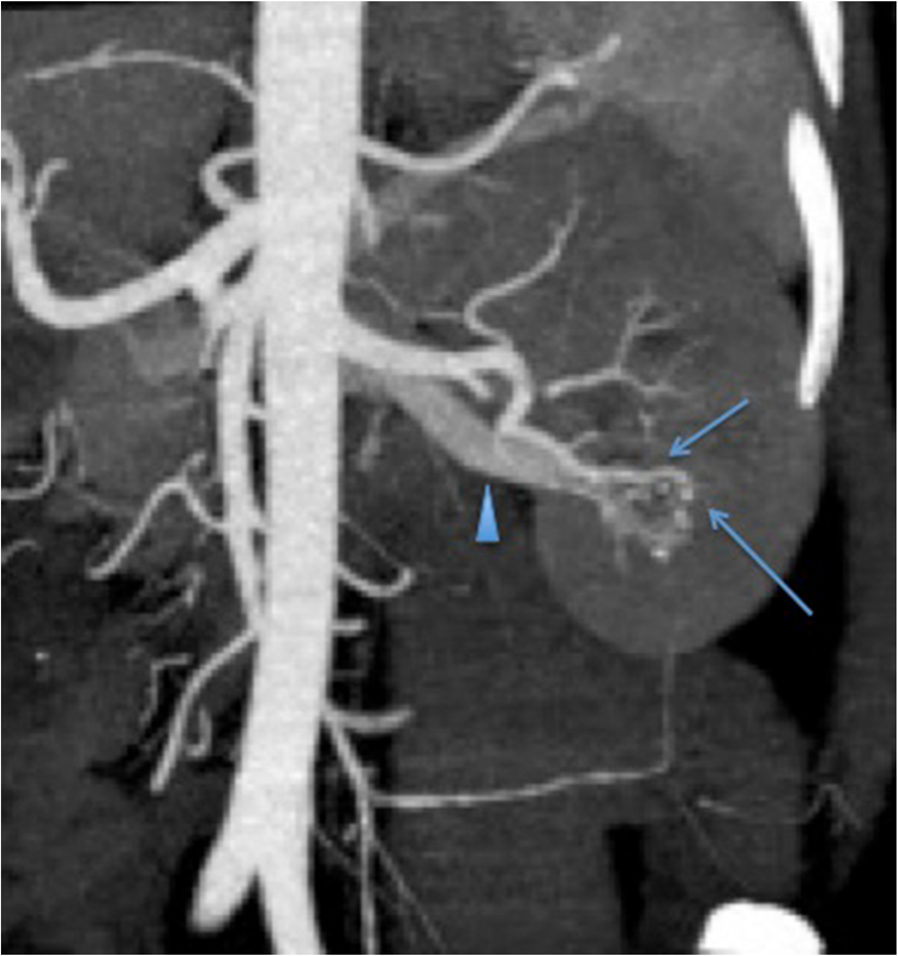
Sagittal oblique multiplanar reformat image of the computed tomography angiogram shows early filling of the left renal vein (arrowhead) and several hypertrophic peripheral renal artery branches in the lower pole of the left kidney (arrows), this is consistent with a renal arteriovenous malformation.

Plain, nephrographic and pyelographic phase computed tomography (CT) imaging of the kidneys was performed, which showed blood clots within the left kidney along with delayed contrast excretion, but failed to demonstrate any cause for the hematuria (Figures 1 and 2). A flexible cystoscopy showed no bladder lesions and no active bleeding from the ureteric orifices. The working diagnosis was then a possible recent passage of a stone with acute pyelovenous leakage and bleeding.

Our patient complained of left flank pain on day 2 of his admission and continued experiencing multiple intermittent episodes of mild to moderate hematuria over a period of about 6 days. Management consisted of continuous bladder irrigation and with this the patient was relatively well and stable. On day 6, our patient had an episode of gross hematuria with acute clot retention. He became symptomatically anemic with a hemoglobin level as low as 8.9g/dL and a hematocrit level of 29.5%, requiring transfusion of a total of 1L of packed cells.

**Figure 3:**
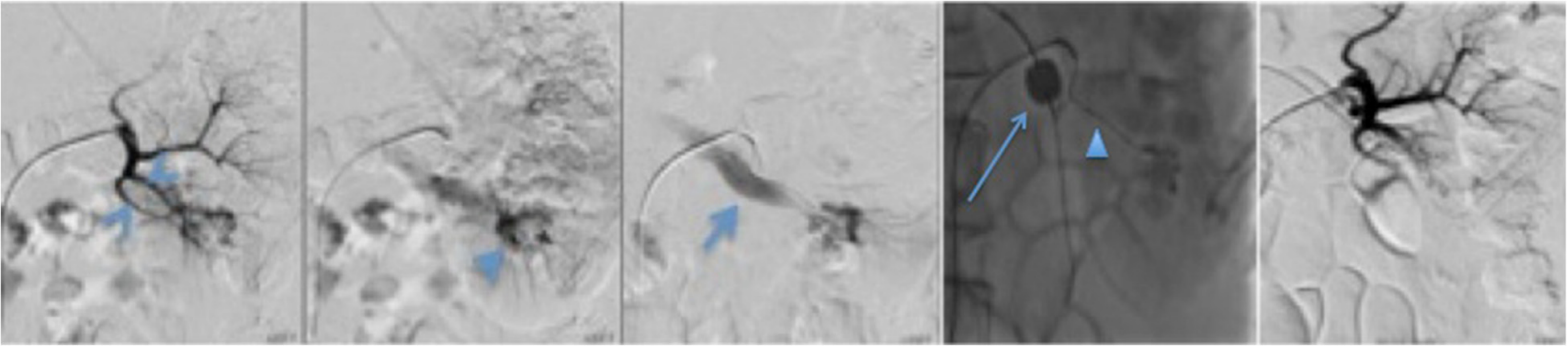
**Selective left renal angiogram demonstrates the hypertrophic peripheral renal artery branches in the lower pole (arrowheads in first panel) with a large nidus (arrowhead in second panel), and rapid shunting to the lower pole renal vein (arrow in the third panel).** A balloon catheter has been placed in the left renal vein to prevent reflux of the embolic agent (arrow in fourth panel), and superselective catheterization of the left lower pole renal artery has been performed using a microcatheter (arrowhead in fourth panel). Selected image after embolization of the renal arteriovenous malformation with absolute alcohol and polyvinyl alcohol particles show truncation of the hypertrophic left lower pole peripheral renal artery branches with absence of shunting to the lower pole renal vein (fifth panel).

A CT angiogram was performed, which demonstrated an AVM in the left lower pole, being fed by a lower polar intersegmental renal artery branch (Figure 2). He underwent arterial embolization of this structure later on the same day. Using a right common femoral artery approach, selective renal angiogram and subsequent superselective left lower pole renal artery catheterization were performed (Figure 3). Embolization was performed using absolute alcohol and lipiodol in a 1:1 ratio and 355 to 500 microns polyvinyl alcohol (PVA) particles (Contour®; Boston Scientific, Natick, MA, USA). He subsequently made a full recovery and was discharged well and stable on day 10 of his admission.

## Discussion

Gross hematuria and flank pain are among the most frequent complaints seen in urology outpatient clinics worldwide. Common causes of gross hematuria include urinary tract infection, urolithiasis and neoplasms of the genitourinary tract. Renal AVM are among the rare causes of gross hematuria. The consideration of this diagnosis early during diagnostic workup was noted to be paramount to management in view of the short chronology of the disease process and the potential for rapid clinical deterioration.

Renal AVM can be idiopathic, congenital or acquired, and are usually differentiated by their angiographic configuration. Acquired renal AVM (more frequently referred to as arteriovenous fistulas) are the most common type (up to 75% of all cases), frequently due to iatrogenic trauma such as renal biopsy or surgery. Other causes include blunt or penetrating trauma, pyelonephritis and renal cell carcinoma. Such cases are characterized in imaging studies as a solitary communication between an artery and a vein. Congenital renal AVM make up about 25% of all cases, and usually present in the third to fourth decade of life. They appear on angiography as lesions resembling angiomas or tortuous veins with multiple communications between arteries and veins, and are sometimes associated with genetic disorders such as hereditary hemorrhagic telangectasia [2]. Idiopathic renal AVM only comprise up to 5% of all cases, with similar angiographic appearance to that of the acquired type in the absence of an apparent cause. Idiopathic renal AVM are thought to be caused by spontaneous erosion or rupture of a renal artery into a neighboring renal vein.

While many renal AVM are diagnosed as incidental imaging findings in the investigation of hypertension or microscopic hematuria, there are few documented cases of patients initially presenting with gross hematuria with clots. This is sometimes associated with colicky pain, tenderness or a feeling of fullness in the flank region. Signs that may raise suspicion of a renal AVM include a continuous bruit or, less specifically, a palpable mass over the flank. Other more infrequent presentations may include symptoms or signs of congestive heart failure from high-output fistulas or hypotension from massive hematuria.

In the case reported, our patient’s presentation with a first episode of exercise-induced hematuria on a background of preexisting hypertension painted a nonspecific picture with regard to the investigation of this episode. The ultimate development of symptoms such as flank pain and recurrent acute bladder tamponade due to clot retention lent more clues to the diagnosis of renal AVM, particularly in the absence of any radiological clues suggestive of other urologic pathology. Urolithiasis (with or without stone passage) can present like the current case with symptoms such as gross hematuria and flank pain, which was thought to be the initial diagnosis for our present case upon radiologic and cystoscopic examination. However, the persistence of symptoms and clinical deterioration in our present case compelled a second diagnostic approach and hence a decision was made to perform CT angiography. The other common differential diagnoses for urinary tract hemorrhage, genitourinary tumors (including malignancies of the bladder, ureter and prostate), can present with similar symptoms and signs, and the lack of radiologic, cytologic and endoscopic findings early in the diagnostic process were key in excluding this as a cause for urinary tract hemorrhage in the present case [3].

Investigation of patients with renal AVM would be directed depending on their presentation and associated complaints. A complete blood count should be included in the investigational profile of a patient who presents with gross hematuria as a means of monitoring blood loss. Coagulation profiles are useful in determining the presence of any contributing coagulopathies. Renal function testing may be indicated in the analysis of hypertensive patients. Urine testing for microscopy, culture and cytology are indicated in patients presenting with hematuria to uncover any underlying infections, glomerulonephritides or malignancy.

Imaging studies remain an important tool in the diagnosis of renal AVMs. Ultrasonography with color duplex studies tend to be the first line of imaging studies used in the diagnosis of renal AVM in some centers. Findings on color duplex ultrasound scans include turbulent blood flow with increased velocity and increased turbulent diastolic flow into the feeding artery or arterial flow moving in opposite directions in a pair of adjacent vessels [4]. CT urography comprising plain, arterial, nephrographic and pyelographic phase images is a standard first-line imaging modality in the context of gross hematuria in many centers. In our center, the current CT urography protocol comprises plain, nephrographic and pyelographic phase images. This is geared toward diagnosis of the more common causes of hematuria such as calculi and tumors; however, its sensitivity is reduced by the absence of an arterial phase study. While digital subtraction angiography remains the gold standard in the diagnosis of AVM, it is not a first-line modality in view of its invasive nature. This modality is only used in the setting of inconclusive noninvasive imaging findings and with the intention to perform endovascular treatment [5]. Magnetic resonance imaging (MRI) and magnetic resonance angiography (MRA) can be useful noninvasive tools in diagnosis, but are more costly and time-consuming than CT angiography.

Principles of management of renal AVM are dependent upon the presentation of the patient. In the case presented, immediate management included stabilization of our patient and urgent bladder decompression and manual bladder irrigation to relieve acute bladder tamponade. In patients presenting with symptomatic anemia and hypovolemia, immediate treatment would mandate the replenishment of blood and fluid reserves.

Conservative treatment including bed rest and symptomatic treatment can be initiated with some degree of safety in some cases of asymptomatic small renal AVM. Spontaneous regression of renal AVM secondary to renal biopsy has been noted, and there have previously been documented cases of spontaneous regression of congenital renal AVM [6]. However, a large number of cases are symptomatic on presentation, necessitating definitive treatment.

Minimally invasive percutaneous transarterial embolization therapy is the first line in the treatment of renal AVM. Embolization can be performed using different materials such as glue, the Onyx® liquid embolic system (Micro Therapeutics, Inc., Irvine, CA, USA), alcohol, gelatin sponges and foams, and PVA particles. Disadvantages of embolization therapy include the risk of nephropathy secondary to the contrast used during radiographic evaluation as well as the possible need for repeat sessions.

Surgical treatment of renal AVM is reserved for cases of large cirsoid malformations, cases intractable to medical and/or endovascular therapy and cases related to malignancy; there have been documented cases of renal AVM requiring urgent nephrectomy [7]. Moreover, nephrectomy has been noted to hold a certain degree of safety in giant renal arteriovenous high-output fistulas, particularly in patients with unfavorable anatomy such as vena caval fistulae [8]. While total nephrectomy would serve as a curative measure [9], long-term complications including the risk of progressive renal impairment must be considered, especially in the setting of preexisting kidney disease and chronic illness. Partial nephrectomy plays a role in the treatment of smaller, polar renal AVM, including those associated with small renal cell carcinomas. Its role continues to be evaluated in this rare entity, particularly with current therapies stressing nephron sparing and preservation of as much renal function as possible. Ligation of feeding vessels has been implemented in the surgical treatment of smaller, more peripheral AVM. However, such a technique is technically challenging and rarely necessary in the advent of current skills in partial nephrectomy [9].

## Conclusions

Congenital AVM is an uncommon abnormal communication between an artery and a vein that causes vascular shunting; while some communicate via small arteriovenous conduit, others are joined via smaller capillary-like pathway or glomus channels. Some genetic vascular diseases, such as hereditary hemorrhagic telangectasia are associated with AVM in multiple anatomic sites, including the brain and brainstem, spine, lung and liver [10]. These disorders can be discovered incidentally or during workup for an unexpected clinical event (such as intracranial hemorrhage in the brain or dyspnea in the lungs [11]). However, lack of vascular differentiation during angiogenesis can also give rise to AVM in other visceral organs such as the kidneys along with more superficial sites, such as the scalp, face and limbs [11,12].

Renal AVMs are a rare entity and only a limited number of cases have been described in the literature. In the case reports of note, presentation can vary between subtle incidental findings to severe hypertension, congestive heart failure and massive hematuria [1]. The patient in this case report presented with exercise-induced hematuria; an atypical presentation for congenital renal AVM. Most common causes of hematuria such as calculi, infection and neoplasms were considered as diagnosis in the initial stages. However, our patient’s episodic relapses of gross hematuria prompted further radiologic investigation for the less common vascular anomalies.

In light of the myriad of presentations and potential morbidity of such an anomaly, it is important to ascertain a definitive diagnosis and initiate treatment as necessary. For incidentally diagnosed AVMs, endovascular therapy has proven effective in asymptomatic patients or patients that are stable on presentation. However, surgical treatment remains a reasonable choice in unstable patients or in patients whose renovascular anatomy is not favorable for endovascular treatment.

## Consent

Written informed consent was obtained from the patient for publication of this case report and any accompanying images. A copy of the written consent is available for review by the Editor-in-Chief of this journal.

## Abbreviations

AVM: Arteriovenous malformation; CT: Computed tomography; MRI: Magnetic resonance imaging; MRA: Magnetic resonance angiography; PVA: Polyvinyl alcohol.

## Competing interests

The authors declare that they have no competing interests.

## Authors’ contributions

ELD was the Medical Officer in charge of the patient’s care and management throughout his admission to our institution. NLG is the patient’s Doctor in Charge of his management during his admission to our institution as well as his subsequent follow-up. TKH is the Interventional Radiologist who performed the selective renal artery embolization for the patient. All authors read and approved the final manuscript.
